# Habitat and peritumoral CT radiomics accurately predict early treatment response to hepatic arterial infusion chemotherapy combined with tyrosine kinase inhibitors and programmed death−1 inhibitors in unresectable hepatocellular carcinoma

**DOI:** 10.3389/fonc.2026.1820483

**Published:** 2026-05-08

**Authors:** Wenyi Bao, Minghang Shen, Kaidi Long, Bo Huang, Zhiqiang Zhang, Peizhi Li

**Affiliations:** 1 Department of Hepatobiliary Surgery, The Second Affiliated Hospital of Chongqing Medical University, Chongqing, China; 2 Department of Radiology, The First Affiliated Hospital of Chongqing Medical University, Chongqing, China; 3 Department of Hepatobiliary Surgery, The Shapingba Hospital, Chongqing University, Chongqing, China

**Keywords:** habitat, HAIC, HCC, radiomics, therapeutic

## Abstract

**Background:**

The pronounced heterogeneity of hepatocellular carcinoma (HCC) leads to variable responses to hepatic arterial infusion chemotherapy combined with tyrosine kinase inhibitors and PD-1 inhibitors (HTP therapy). This study aimed to develop and validate a combined model integrating habitat radiomics, peritumoral radiomics, and clinical factors to predict early treatment response in patients with unresectable HCC receiving HTP therapy.

**Methods:**

This retrospective two-center study enrolled 213 patients with unresectable HCC who received HTP therapy. Patients from Center 1 (n = 143) were randomly allocated to a training cohort (n = 100) and an internal validation cohort (n = 43); patients from Center 2 (n = 70) served as an independent external testing cohort. Regions of interest from portal venous phase images were partitioned into three habitat subregions via K-means clustering and expanded at thicknesses of 3 mm and 9 mm. Following feature extraction and selection, multiple machine learning algorithms were compared. A combined model was constructed by integrating the habitat model, the best peritumoral model, and clinical risk factors.

**Results:**

Among the 213 treated patients, 109 achieved complete or partial response, yielding an objective response rate of 51.2%. The Habitat model demonstrated superior predictive performance compared to other single models, with AUCs of 0.921, 0.883, and 0.826 in the training, internal validation, and external testing cohorts. The combined model yielded even higher AUCs of 0.955, 0.968, and 0.893 across the three cohorts. Decision curve analysis and calibration curves further confirmed that the combined model provided high net clinical benefit and exhibited good calibration.

**Conclusion:**

The combined model, integrating habitat and peritumoral radiomics with clinical factors, accurately predicts early treatment response to HTP therapy in unresectable HCC. Moreover, the model-derived risk stratification significantly differentiated progression-free survival, underscoring its potential prognostic utility and offering a non-invasive tool to facilitate personalized treatment decisions.

## 1 Introduction

Hepatocellular carcinoma (HCC) is the predominant histological subtype of primary liver cancer, accounting for approximately 75%–85% of all cases. It ranks as the sixth most commonly diagnosed cancer and the third leading cause of cancer-related mortality worldwide (1). Owing to the insidious nature of its early symptoms, most patients are diagnosed at intermediate or advanced stages (e.g., Barcelona Clinic Liver Cancer [BCLC] stage B or C), thereby precluding the opportunity for curative surgical resection (2). Systemic therapies based on tyrosine kinase inhibitors (TKIs) and programmed death-1 (PD-1) inhibitors have become cornerstone treatments for advanced HCC, demonstrating significant survival benefits in pivotal clinical trials such as IMbrave150 and ORIENT-32 (3, 4). Building upon this foundation, combining systemic therapy with locoregional arterial approaches—including transarterial chemoembolization (TACE) and hepatic arterial infusion chemotherapy (HAIC)—can further enhance tumor control (5). A randomized phase III trial demonstrated that HAIC with oxaliplatin, fluorouracil, and leucovorin (HAIC-FOLFOX), which enables sustained delivery of high-dose chemotherapy to the tumor site, significantly improved both overall survival and progression-free survival compared with TACE in patients with unresectable HCC (6). Currently, the triple-therapy regimen comprising HAIC-FOLFOX, TKIs, and PD-1 inhibitors has shown promising clinical prospects in the management of advanced HCC in China (7, 8). However, due to the marked cellular heterogeneity of HCC, treatment responses vary substantially across individuals (9). Therefore, the non-invasive identification of patients most likely to benefit from such intensive therapy prior to treatment initiation is of critical importance for guiding personalized therapeutic strategies and improving clinical outcomes.

Radiomics has been extensively applied in oncology for tumor differentiation, prognosis prediction, and treatment evaluation, among other uses, offering a non-invasive window into tumor biology (10–13). However, conventional radiomics typically reduces the tumor to a homogeneous volume, thereby obscuring spatial variation among functionally distinct subregions. (14, 15). In contrast, habitat imaging—a method that partitions the tumor into multiple distinct subregions, termed habitats, based on similarity in imaging features—enables fine-grained quantification of the spatial architecture of intratumoral heterogeneity. Each habitat represents an area with shared imaging characteristics, reflecting its specific cellular composition and microenvironmental conditions (16). This technique has demonstrated considerable value in predicting treatment response across various malignancies, including liver, thyroid, and breast cancers (17–19). However, existing studies applying habitat models to predict treatment response in hepatocellular carcinoma have primarily concentrated on intratumoral spatial heterogeneity, often neglecting the prognostically valuable peritumoral region. This region represents a critical interface where tumor cells interact with the adjacent liver parenchyma, driving processes such as invasion, immune modulation, and metabolic reprogramming. Importantly, the imaging phenotype of the peritumoral area exhibits substantial heterogeneity, which has been shown to harbor significant prognostic and predictive information (20, 21).

Accordingly, the present study aims to develop a multidimensional integrated predictive model by combining habitat radiomics, peritumoral radiomics, and clinical risk factors, with the objective of enabling non-invasive and accurate pretreatment prediction of early treatment response and progression-free survival (PFS) in patients with unresectable HCC receiving HAIC-FOLFOX combined with TKIs and PD-1 inhibitors (HTP) therapy.

## 2 Materials and methods

### 2.1 Patient population

This retrospective study was approved by the Ethics Committees of the two participating institutions: The Second Affiliated Hospital of Chongqing Medical University (Center 1) and The First Affiliated Hospital of Chongqing Medical University (Center 2). Given that all patient data were fully de-identified prior to analysis, the requirement for informed consent was waived.

A total of 562 patients with HCC who received HAIC-FOLFOX combined with TKIs and PD-1 inhibitors at Center 1, and 285 patients from Center 2 between December 2021 and December 2024, were initially enrolled. The inclusion criteria were as follows (1): age ≥ 18 years; (2) Child-Pugh grade A or B liver function; (3) a pathological or clinical diagnosis of intermediate- or advanced-stage HCC (BCLC stage B or C); (4) treatment with HAIC-FOLFOX combined with TKIs and PD-1 inhibitors, with HAIC as the initial treatment modality. Exclusion criteria included: (1) absence of pre- or post-treatment contrast-enhanced CT images; (2) poor image quality or incomplete clinical data; (3) presence of other primary malignancies; (4) any prior anti-tumor therapy before HAIC.

**Figure 1:**
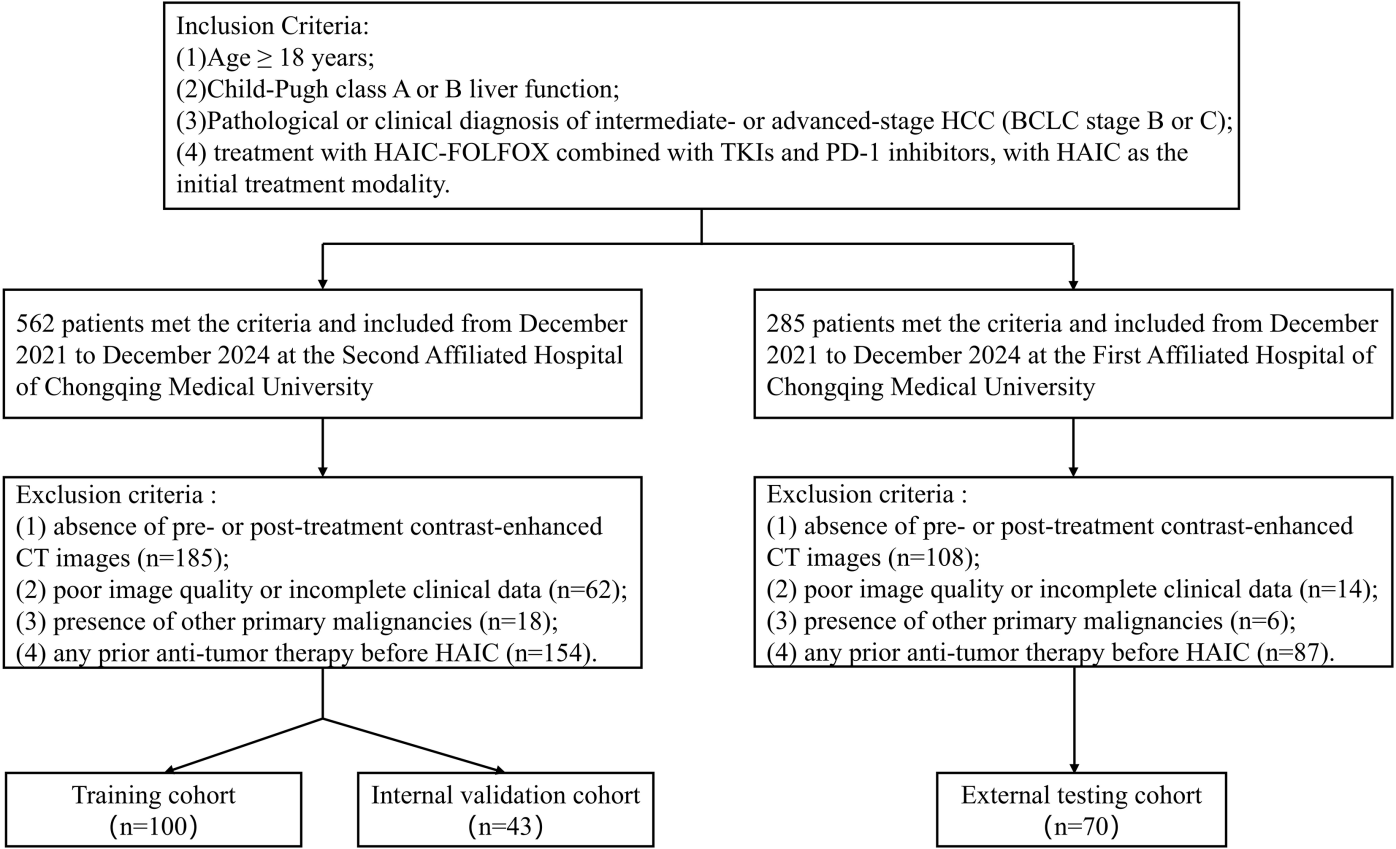
Flowchart of patient selection and cohort allocation.

Following the application of the exclusion criteria, 143 eligible patients from Center 1 were ultimately included and randomly assigned in a 7:3 ratio to a training cohort (n = 100) and an internal validation cohort (n = 43). Seventy patients from Center 2 served as an independent external testing cohort. Patient selection flowchart is shown in **Figure 1**.

### 2.2 Baseline evaluation

The following baseline clinical characteristics were collected for each patient: (1) Demographic characteristics: sex, age, history of hepatitis B, history of cirrhosis, presence of ascites, body mass index (BMI), and Eastern Cooperative Oncology Group (ECOG) performance status; (2) Laboratory parameters measured within one week prior to HAIC-FOLFOX: neutrophil count (Neu), lymphocyte count (Ly), platelet count (PLT), alpha-fetoprotein (AFP), alanine aminotransferase (ALT), aspartate aminotransferase (AST), alkaline phosphatase (ALP), gamma-glutamyl transferase (GGT), total bilirubin (TBIL), albumin (ALB), prothrombin time (PT), ALBI score and grade, Child-Pugh class, and BCLC stage; (3) Tumor characteristics: number of lesions, maximum tumor diameter, presence of portal vein tumor thrombus, vascular invasion, and distant metastasis.

### 2.3 Hepatic arterial infusion chemotherapy

Under digital subtraction angiography (DSA) guidance, arterial access was obtained via the femoral artery using the Seldinger technique. A 5-French catheter was then advanced over a guidewire into the celiac trunk, common hepatic artery, and proper hepatic artery for angiographic evaluation of the arterial supply. Subsequently, a 2.7-French microcatheter was superselectively positioned within the target feeding vessel. The external portion of the microcatheter was covered with sterile gauze and secured to the thigh skin with medical adhesive tape.

All chemotherapeutic agents were administered via hepatic arterial infusion through the microcatheter according to the following FOLFOX-based regimen: oxaliplatin 85 mg/m² (infused over 0–2 hours on day 1), leucovorin 400 mg/m² (infused over 2–3 hours on day 1), followed by a bolus of 5-fluorouracil (5-FU) 400 mg/m² (administered over 3–4 hours on day 1) and a subsequent continuous infusion of 5-FU at 2400 mg/m² over 46 hours. The treatment cycle was repeated every 3–4 weeks, with identical protocols and dosages applied to patients at both centers.

### 2.4 TKIs and PD-1 inhibitors

TKI therapy was initiated within one week after the first HAIC procedure. The regimens included sorafenib (400 mg twice daily) and lenvatinib (8 mg once daily for patients weighing ≤60 kg, or 12 mg once daily for those weighing >60 kg). PD-1 inhibitors were administered intravenously either immediately after or within one week following the procedure, using either sintilimab (200 mg) or camrelizumab (200 mg). In the event of intolerable toxicity or confirmed disease progression, dose reduction, temporary interruption, or a switch to second-line agents was considered.

### 2.5 CT imaging protocols

All patients underwent a four-phase contrast-enhanced CT (CECT) protocol using either a SOMATOM Force or SOMATOM Definition Flash scanner (both Siemens Healthineers, Germany). The protocol comprised non-contrast, arterial (AP), portal venous (PVP), and delayed (DP) phases, with full coverage of the liver. Scanning parameters were as follows: tube voltage, 120 kVp; tube current modulated using automatic tube current modulation; matrix, 512 × 512; slice thickness, 5 mm. Following the non-contrast acquisition, a contrast agent (iohexol, 300 mg/mL; GE Healthcare) was administered via an antecubital vein using a power injector at a rate of 3 mL/s, with a total dose of 1.5–2 mL/kg body weight. AP, PVP, and DP images were acquired with delays of 25–30 s, 60–70 s, and 150–180 s after the start of contrast injection, respectively.

### 2.6 Tumor segmentation and response assessment

Preoperative portal venous phase CT images of all patients were stored in DICOM format and subsequently converted to NIFTI format. All images were resampled to a uniform voxel size of 1 × 1 × 1 mm³, and the window width and level were standardized to 200 Hounsfield units (HU) and 45 HU, respectively. Regions of interest (ROI) were collaboratively delineated on all images by two radiologists, each with over 10 years of experience in abdominal imaging, using ITK-SNAP software (version 3.8.0, http://www.itksnap.org). The tumor boundaries were manually contoured slice-by-slice to generate the volume of interest (VOI) for each patient. In cases of disagreement, a consensus was reached through arbitration by a third senior radiologist with more than 20 years of experience.

**Figure 2:**
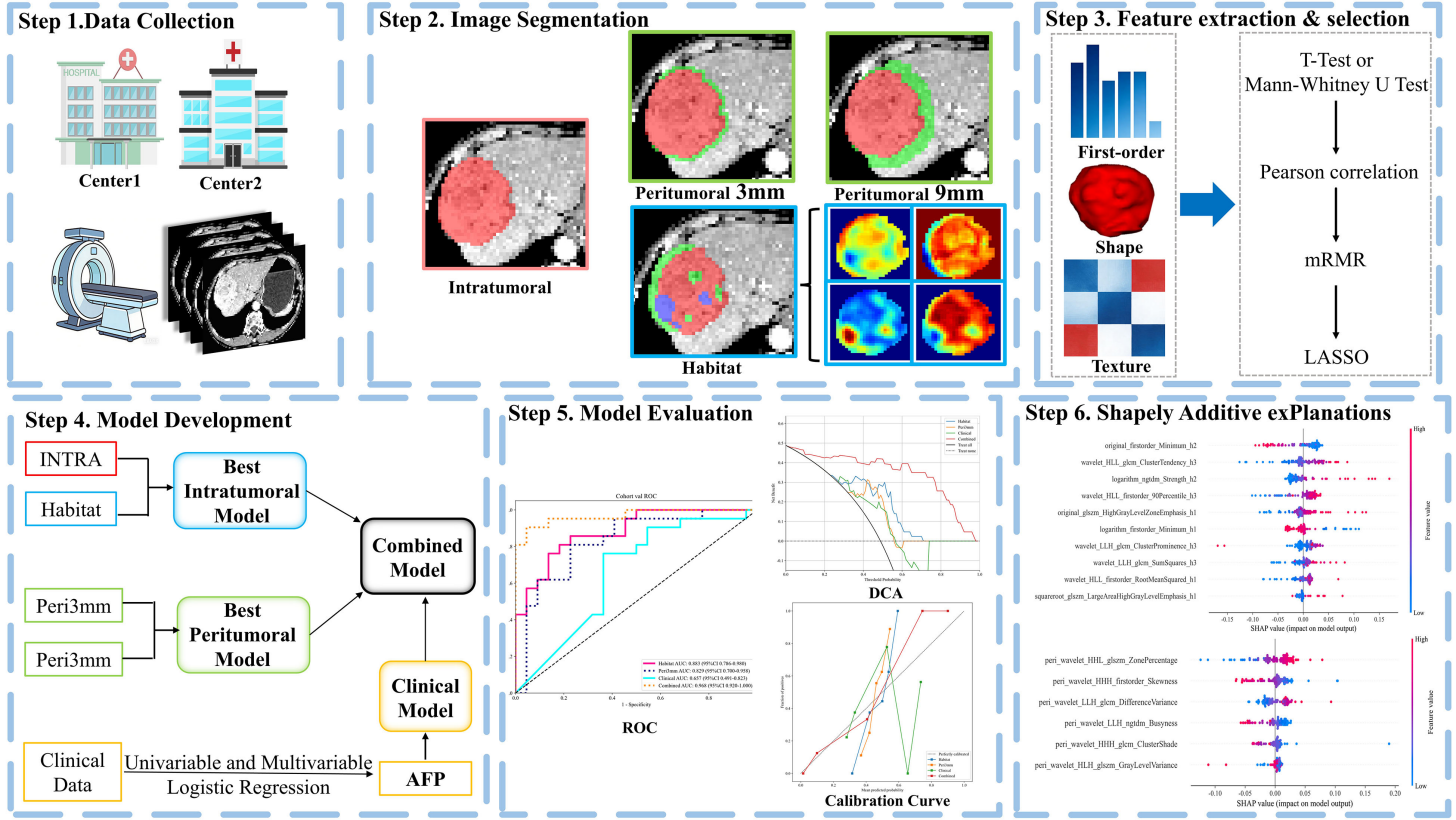
Overall workflow of the study.

According to the modified Response Evaluation Criteria in Solid Tumors (mRECIST) (22), treatment response was assessed using contrast-enhanced CT performed 1–2 months following the first HAIC treatment. The definitions were as follows: Complete response (CR) was defined as the absence of intratumoral arterial enhancement in all target lesions on contrast-enhanced CT. Partial response (PR) was defined as at least a 30% decrease in the sum of the diameters of target lesions, measured in the arterial phase. Progressive disease (PD) was defined as at least a 20% increase in the sum of the diameters of target lesions (arterial phase) and/or the appearance of new lesions. Stable disease (SD) was defined as changes in target lesions that did not meet the criteria for either PR or PD. Accordingly, patients were categorized into two groups: the objective response group (ORR+), comprising patients who achieved CR or PR, and the non-objective response group (ORR–), comprising patients with SD or PD. The enrolled patients were followed up every 3–4 weeks, and contrast-enhanced CT or MRI was performed to evaluate treatment response. The endpoint of follow-up was set for June 10, 2025. Progression-free survival (PFS) was defined as the time from treatment initiation to disease progression, death, or the cut-off date. The study flow is illustrated in **Figure 2**.

### 2.7 Peritumoral region dilation

To investigate the influence of the peritumoral region, the original tumor ROI mask was systematically expanded outward to create two concentric peritumoral shells at radial distances of 3 mm and 9 mm from the tumor margin, using the mask inpainting toolkit from the Onekey AI platform. Manual correction was applied to areas where the expanded region extended beyond the liver boundary or encompassed major intrahepatic vessels, bile ducts, or other critical anatomical structures.

### 2.8 Habitat generation

Local features, including entropy and energy, were extracted from each voxel within the volume of interest (VOI). A 19-dimensional feature vector was generated for each image block using a 3 × 3 × 3 non-overlapping sliding window. The optimal number of clusters was determined by evaluating the Calinski–Harabasz index across a range of cluster numbers (k = 2 to 10). Subsequently, K-means clustering was applied to partition each tumor sample into distinct subregions based on the resulting feature vectors.

### 2.9 Feature extraction and selection

In accordance with the Imaging Biomarker Standardization Initiative guidelines, handcrafted radiomic features were extracted using the Pyradiomics toolbox (version 3.0.1) from the following ROIs: (1) the intratumoral region; (2) each of the habitat subregions; (3) the peritumoral region expanded by 3 mm; and (4) the peritumoral region expanded by 9 mm. A total of 1,561 features were extracted per ROI; thus, the total number of habitat-derived features was 1,561 multiplied by the number of subregions. The extracted features encompassed three categories: (I) shape-based features describing three-dimensional geometric properties; (II) first-order intensity features capturing voxel value distributions; and (III) texture features derived from matrices including the gray-level co-occurrence matrix (GLCM), gray-level run-length matrix (GLRLM), gray-level size zone matrix (GLSZM), gray-level dependence matrix (GLDM), and neighboring gray-tone difference matrix (NGTDM).

All extracted features were standardized using Z-score normalization. Features showing a significant association with treatment response (p < 0.05) were identified using the independent samples t-test or the Mann-Whitney U test, as appropriate for the data distribution. To mitigate multicollinearity, Pearson correlation coefficients were calculated; for any pair of features with a correlation coefficient exceeding 0.9, one feature from the pair was excluded. A greedy recursive elimination strategy was subsequently applied to further reduce feature redundancy. For each region of interest, the top 15 features were then preselected using the minimum redundancy maximum relevance (mRMR) algorithm. Finally, the least absolute shrinkage and selection operator (LASSO) regression model with 10-fold cross-validation was employed to determine the optimal regularization parameter (λ) and to establish the final set of non-zero coefficient features. These features, along with their respective LASSO coefficients, constituted the radiomics signature for each region. The derived signatures were subsequently used for model construction.

### 2.10 Model development

Based on the radiomics signatures constructed for each region, four prediction models were developed according to the distinct regions of interest: intratumoral (INTRA), peritumoral 3 mm (Peri3mm), peritumoral 9 mm (Peri9mm), and habitat (Habitat). For each model, four machine learning algorithms—Random Forest, XGBoost, Multilayer Perceptron (MLP), and Extra Trees—were employed. To determine the optimal hyperparameters and prevent overfitting, we employed a five-fold cross-validation strategy combined with a grid search, performed exclusively within the training cohort. Using these optimal parameters, the final model for each algorithm was then retrained on the entire training cohort. The algorithm achieving the highest area under the curve (AUC) in the internal validation cohort was selected as the optimal model for the corresponding feature set. To address class imbalance, the Synthetic Minority Over-sampling Technique (SMOTE) was applied to the training cohort during model training. Finally, a clinical prediction model was constructed using logistic regression based on the clinical risk factors identified by univariable and multivariable logistic regression analyses.

To further improve predictive performance, the best intratumoral radiomics model and the best peritumoral radiomics model were identified by comparing their performance in the internal validation cohort, using the AUC as the primary criterion, with accuracy and other metrics serving as secondary references. The predicted probabilities from these two selected radiomics models, together with the predicted probability from the clinical model, were then used as input features to train a secondary logistic regression model, which constituted the final combined model. To evaluate model stability, a repeated five-fold cross-validation was performed on the training cohort with stratified sampling by ORR status.

### 2.11 Model evaluation and interpretation

The discriminative performance of the models was assessed using receiver operating characteristic (ROC) curve analysis, with the area under the curve (AUC) as the primary metric. The DeLong test was used to compare AUC values between models and to determine statistical significance. The integrated discrimination improvement (IDI) and net reclassification index (NRI) were calculated to evaluate the incremental predictive value of the models. Calibration curves were plotted to assess model goodness-of-fit, and decision curve analysis (DCA) was performed to evaluate clinical utility. To enhance interpretability, SHAP (SHapley Additive exPlanations) analysis was conducted, generating beeswarm plots to illustrate global feature importance and force plots to visualize individual predictions.

### 2.12 Survival analysis

To evaluate the prognostic value of the combined model, patients were stratified into high-risk and low-risk groups based on the optimal cutoff value determined by the maximum Youden index method. Kaplan–Meier survival curves were generated to compare progression-free survival (PFS) between the two groups, and the log-rank test was used to assess the statistical significance of the differences.

### 2.13 Statistical analysis

The following statistical methods were employed to compare clinical characteristics among different patient groups. For continuous variables, the independent samples t-test was used for inter-group comparisons; when the assumptions of the t-test were violated, the non-parametric Mann–Whitney U test was applied instead. For categorical variables, frequencies and percentages were reported, and comparisons were performed using the chi-square (χ²) test or Fisher’s exact test, as appropriate based on the dataset. All statistical analyses were performed using R software (version 4.2.2) to ensure reproducibility and reliability. A two-tailed p-value < 0.05 was considered statistically significant.

## 3 Results

### 3.1 Baseline characteristics

A total of 213 patients with unresectable HCC who received HTP therapy were included in this study. The overall objective response rate (ORR) was 51.2%, with ORRs of 54.0%, 51.2%, and 47.1% observed in the training cohort, internal validation cohort, and external testing cohort, respectively. Detailed baseline clinical characteristics of the patients are summarized in **Table 1**. In the training cohort, significant differences were observed between the ORR+ and ORR− groups with respect to performance status (P = 0.001) and serum AFP level (P < 0.001). In the external testing cohort, age (P = 0.037) and prothrombin time (P = 0.003) demonstrated statistically significant between-group differences. No significant differences were found for the remaining variables across all cohorts.

**Table 1:** Baseline demographic and clinical characteristics of patients.

Variables	Training cohort (*n* = 100)		*p* value	Internal validation cohort (*n* = 43)		*p* value	External testing cohort (*n* = 70)		*p* value
	ORR+	ORR-		ORR+	ORR-		ORR+	ORR-	
Age (years)	56.72 ± 11.95	57.13 ± 11.56	0.863	59.55 ± 11.75	56.62 ± 9.32	0.372	51.12 ± 8.78	55.95 ± 10.06	0.037
BMI	22.69 ± 3.27	23.66 ± 4.63	0.474	21.93 ± 2.31	22.56 ± 3.22	0.680	23.95 ± 6.22	23.04 ± 2.67	0.707
Gender			0.778			0.634			0.462
Female	5(9.26)	6(13.04)		3(13.64)	1(4.76)		4(12.12)	8(21.62)	
Male	49(90.74)	40(86.96)		19(86.36)	20(95.24)		29(87.88)	29(78.38)	
HBV			0.249			0.782			0.779
Absence	9(16.67)	13(28.26)		6(27.27)	4(19.05)		2(6.06)	4(10.81)	
Presence	45(83.33)	33(71.74)		16(72.73)	17(80.95)		31(93.94)	33(89.19)	
Cirrhosis			0.866			0.902			0.591
Absence	25(46.30)	23(50.00)		8(36.36)	9(42.86)		13(39.39)	18(48.65)	
Presence	29(53.70)	23(50.00)		14(63.64)	12(57.14)		20(60.61)	19(51.35)	
Ascites			0.233			1			0.566
Absence	38(70.37)	38(82.61)		18(81.82)	17(80.95)		29(87.88)	35(94.59)	
Presence	16(29.63)	8(17.39)		4(18.18)	4(19.05)		4(12.12)	2(5.41)	
PS			0.001			1			0.583
ECOG 0	26(48.15)	7(15.22)		2(9.09)	2(9.52)		5(15.15)	3(8.11)	
ECOG 1	28(51.85)	39(84.78)		20(90.91)	19(90.48)		28(84.85)	34(91.89)	
ALBI score	-2.45 ± 0.48	-2.48 ± 0.57	0.785	-2.50 ± 0.46	-2.42 ± 0.44	0.528	-2.38 ± 0.47	-2.52 ± 0.45	0.207
ALBI grade			0.807			0.407			0.493
1	19(35.19)	19(41.30)		10(45.45)	6(28.57)		11(33.33)	15(40.54)	
2	34(62.96)	26(56.52)		12(54.55)	15(71.43)		21(63.64)	22(59.46)	
3	1(1.85)	1(2.17)		0	0		1(3.03)	0	
Child Pugh grade			0.684			1			0.199
A	43(79.63)	39(84.78)		18(81.82)	17(80.95)		30(90.91)	37(100.00)	
B	11(20.37)	7(15.22)		4(18.18)	4(19.05)		3(9.09)	0	
Tumor size (cm)	8.28 ± 3.97	8.67 ± 3.39	0.566	9.54 ± 3.13	9.64 ± 4.26	0.926	8.34 ± 3.45	8.61 ± 3.81	0.759
Number of tumors			0.268			0.902			0.708
<2	27(50.00)	17(36.96)		14(63.64)	12(57.14)		11(33.33)	15(40.54)	
≥2	27(50.00)	29(63.04)		8(36.36)	9(42.86)		22(66.67)	22(59.46)	
VP			0.452			0.210			0.158
0	21(38.89)	23(50.00)		10(45.45)	4(19.05)		15(45.45)	17(45.95)	
1	0	1(2.17)		0	0		0	0	
2	3(5.56)	4(8.70)		4(18.18)	5(23.81)		0	5(13.51)	
3	21(38.89)	12(26.09)		5(22.73)	10(47.62)		10(30.30)	8(21.62)	
4	9(16.67)	6(13.04)		3(13.64)	2(9.52)		8(24.24)	7(18.92)	
Vascular invasion			0.765			0.892			1
Absence	29(53.70)	27(58.70)		13(59.09)	11(52.38)		23(69.70)	25(67.57)	
Presence	25(46.30)	19(41.30)		9(40.91)	10(47.62)		10(30.30)	12(32.43)	
Metastasis			0.354			0.270			0.952
Absence	43(79.63)	32(69.57)		16(72.73)	19(90.48)		27(81.82)	29(78.38)	
Presence	11(20.37)	14(30.43)		6(27.27)	2(9.52)		6(18.18)	8(21.62)	
BCLC			0.148			0.355			0.704
B	16(29.63)	21(45.65)		8(36.36)	4(19.05)		14(42.42)	13(35.14)	
C	38(70.37)	25(54.35)		14(63.64)	17(80.95)		19(57.58)	24(64.86)	
AFP (ng/ml)	301.19 ± 472.40	703.57 ± 476.71	<0.001	516.18 ± 537.26	777.84 ± 446.74	0.072	579.65 ± 556.73	817.77 ± 534.23	0.084
ALB (g/L)	38.16 ± 5.56	38.87 ± 5.66	0.530	38.71 ± 4.63	38.17 ± 4.39	0.699	37.37 ± 5.51	38.73 ± 4.96	0.281
Neu (10^9/L)	3.77 ± 2.64	4.30 ± 5.26	0.779	3.76 ± 2.93	3.36 ± 2.35	0.504	3.87 ± 1.99	4.04 ± 1.83	0.451
Ly (10^9/L)	1.51 ± 1.05	1.56 ± 1.80	0.825	1.03 ± 0.41	1.26 ± 1.53	0.465	1.03 ± 0.42	1.14 ± 0.42	0.219
PLT (10^9/L)	159.24 ± 76.80	161.91 ± 83.08	0.986	153.64 ± 78.48	150.24 ± 71.38	0.883	153.12 ± 65.20	183.49 ± 90.01	0.208
ALT (U/L)	53.02 ± 34.91	63.59 ± 50.29	0.358	61.68 ± 62.48	63.76 ± 56.63	0.770	53.42 ± 21.64	59.95 ± 56.35	0.306
AST (U/L)	85.54 ± 71.97	83.57 ± 70.80	0.771	98.77 ± 82.63	116.86 ± 100.21	0.671	86.09 ± 66.27	86.24 ± 65.82	0.995
ALP (U/L)	166.89 ± 89.39	172.89 ± 86.84	0.587	172.41 ± 110.71	177.00 ± 70.74	0.337	130.06 ± 53.91	160.22 ± 113.55	0.601
GGT (U/L)	187.57 ± 138.82	259.41 ± 254.31	0.213	221.70 ± 238.40	223.43 ± 134.50	0.307	199.45 ± 159.98	228.30 ± 276.36	0.356
TBIL (umol/L)	17.83 ± 8.36	55.93 ± 253.50	0.961	17.89 ± 10.37	21.48 ± 15.77	0.536	18.10 ± 9.02	17.07 ± 9.46	0.300
PT (s)	14.01 ± 1.33	13.58 ± 1.06	0.092	14.16 ± 1.23	13.92 ± 1.44	0.644	14.54 ± 1.12	13.72 ± 1.13	0.003

Unless otherwise noted, data are shown as number of patients, with the percentage in parentheses. Continuous variables are presented as mean ± SD. AFP, alpha-fetoprotein; ALB, albumin; ALBI, albumin-bilirubin grade; ALP, alkaline phosphatase; ALT, alanine aminotransferase; AST, aspartate aminotransferase; BCLC, Barcelona Clinic Liver Cancer stage; BMI, body mass index; GGT, gamma-glutamyl transferase; HBV, hepatitis B virus; Neu, neutrophil count; Ly, lymphocyte count; PLT, platelet count; PS, performance status; PT, prothrombin time; TBIL, total bilirubin; VP, vascular invasion.

### 3.2 Habitat analysis

**Figure 3:**
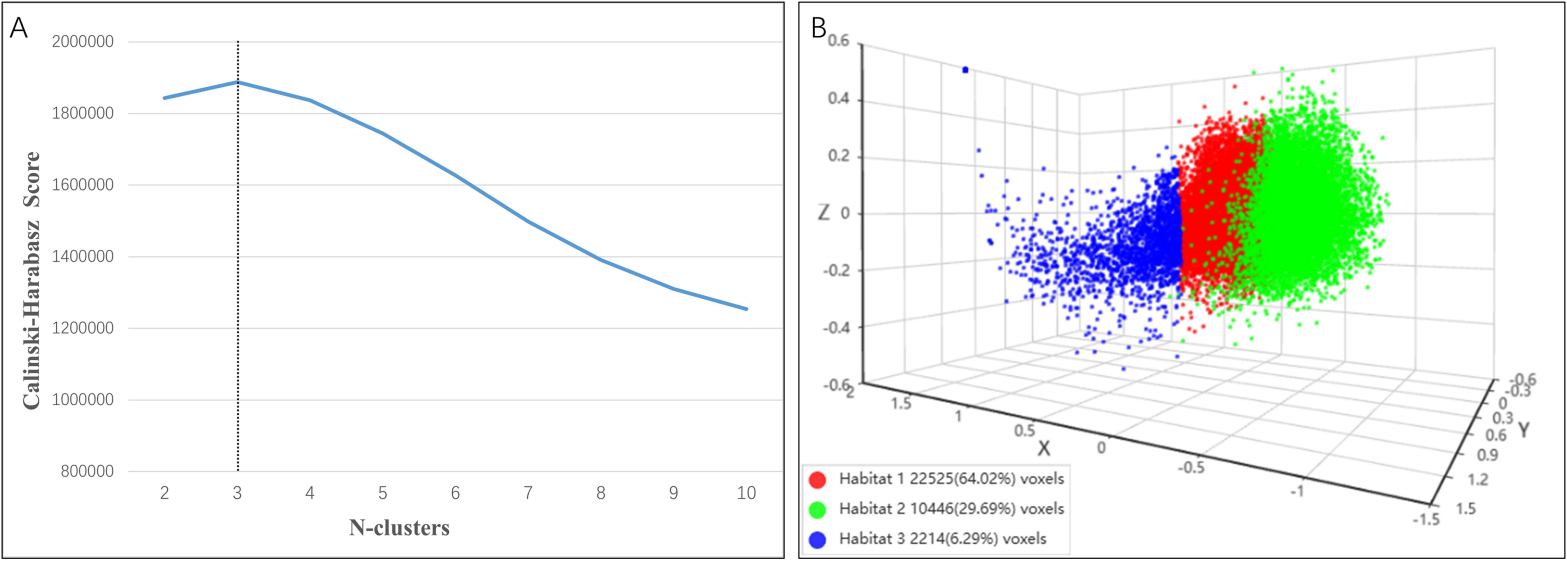
Habitat clustering results. **(A)** Calinski–Harabasz score plot. The vertical dashed line indicates the optimal cluster number (k = 3), corresponding to the peak score. **(B)** Three-dimensional visualization of the identified clusters. Voxel counts and proportional volumes of each habitat subregion (Habitat 1, 2, and 3) are provided in the legend.

Determined by the Calinski–Harabasz index, the optimal number of clusters was three (**Figure 3A**). The voxel counts and proportional volumes of the three resulting subregions are presented in **Figure 3B**, with the following distribution: Habitat 1 (64.02%), Habitat 2 (29.69%), and Habitat 3 (6.29%). Detailed descriptions of the 19 local features used to characterize each habitat region are provided in the **Supplementary Data**.

### 3.3 Performance comparison of radiomics models

After a multi-step feature selection process (**Supplementary Figure 1**), the final numbers of retained features for each radiomics model were as follows: INTRA, 9 features; Habitat, 10 features; Peri3mm, 6 features; and Peri9mm, 3 features (**Supplementary Figure 2**). Based on the highest area under the curve (AUC) achieved in the internal validation cohort, the optimal algorithm for each model was determined: Extra Trees for INTRA, Habitat, and Peri3mm, and Random Forest for Peri9mm. Comparative ROC curves of the different algorithms are provided in **Supplementary Figure 3**.

**Table 2** summarizes the predictive performance of all six models across the three cohorts. In the training cohort, the Habitat model achieved an AUC of 0.921 (95% CI: 0.871–0.971), outperforming the INTRA model (AUC = 0.829, 95% CI: 0.749–0.908). This superior performance was maintained in the internal validation cohort (Habitat: 0.883 vs. INTRA: 0.699) and the external testing cohort (Habitat: 0.826 vs. INTRA: 0.712). Moreover, the Habitat model demonstrated higher accuracy and sensitivity than the INTRA model across all three cohorts. Consequently, the Habitat model was selected as the optimal intratumoral radiomics model.

**Table 2:** The performance of the six models across the training, internal validation, and external testing cohorts.

Model	Cohort	Accuracy	AUC	95%CI	Sensitivity	Specificity	PPV	NPV
INTRA	Training	0.790	0.829	0.749 - 0.908	0.761	0.815	0.778	0.800
	Internal validation	0.674	0.699	0.541 - 0.857	0.524	0.818	0.733	0.643
	External testing	0.686	0.712	0.590 - 0.833	0.730	0.636	0.692	0.677
Habitat	Training	0.840	0.921	0.871 - 0.971	0.935	0.759	0.768	0.932
	Internal validation	0.814	0.883	0.786 - 0.980	0.857	0.773	0.783	0.850
	External testing	0.786	0.826	0.730 - 0.923	0.865	0.697	0.762	0.821
Peri3mm	Training	0.800	0.866	0.797 - 0.935	0.804	0.796	0.771	0.827
	Internal validation	0.791	0.829	0.701 - 0.958	0.810	0.773	0.773	0.810
	External testing	0.714	0.748	0.632 - 0.863	0.622	0.818	0.793	0.659
Peri9mm	Training	0.750	0.804	0.718 - 0.890	0.652	0.769	0.738	0.769
	Internal validation	0.698	0.649	0.479 - 0.820	0.667	0.727	0.700	0.696
	External testing	0.671	0.651	0.519 - 0.783	0.703	0.636	0.684	0.656
Clinical	Training	0.740	0.725	0.621 - 0.828	0.783	0.704	0.692	0.792
	Internal validation	0.698	0.657	0.491 - 0.823	0.762	0.636	0.667	0.737
	External testing	0.629	0.612	0.487 - 0.737	0.676	0.576	0.641	0.613
Combined	Training	0.920	0.955	0.920 - 0.991	0.848	0.981	0.975	0.883
	Internal validation	0.930	0.968	0.920 - 1.000	0.905	0.955	0.950	0.913
	External testing	0.800	0.893	0.822 - 0.964	0.676	0.939	0.926	0.721

AUC, area under the receiver operating characteristic curve; CI, confidence interval; PPV, positive predictive value; NPV, negative predictive value.

The Peri3mm model yielded AUCs of 0.866 (95% CI: 0.797–0.935), 0.829 (95% CI: 0.701–0.958), and 0.748 (95% CI: 0.632–0.863) in the training, internal validation, and external testing cohorts, respectively, all higher than those of the Peri9mm model (0.804, 0.649, and 0.651, respectively). Meanwhile, coupled with its superior accuracy and sensitivity in the internal validation cohort, the Peri3mm model was selected as the optimal peritumoral radiomics model.

### 3.4 Feature importance interpreted by SHAP analysis

SHAP analysis provides an intuitive visualization of the global importance ranking of radiomic features and the relationship between feature values and their impact on predictions. In the SHAP beeswarm plot, radiomic features are arranged along the vertical axis in descending order of global importance, while the horizontal axis represents the SHAP value, which quantifies the direction and magnitude of each feature’s contribution to predicting HTP treatment response. Each dot corresponds to the SHAP value of a specific feature for an individual patient; dots are distributed horizontally and stacked vertically to reflect the density of samples with similar SHAP values. Dot color indicates feature value (blue: low; red: high).

**Figure 4:**
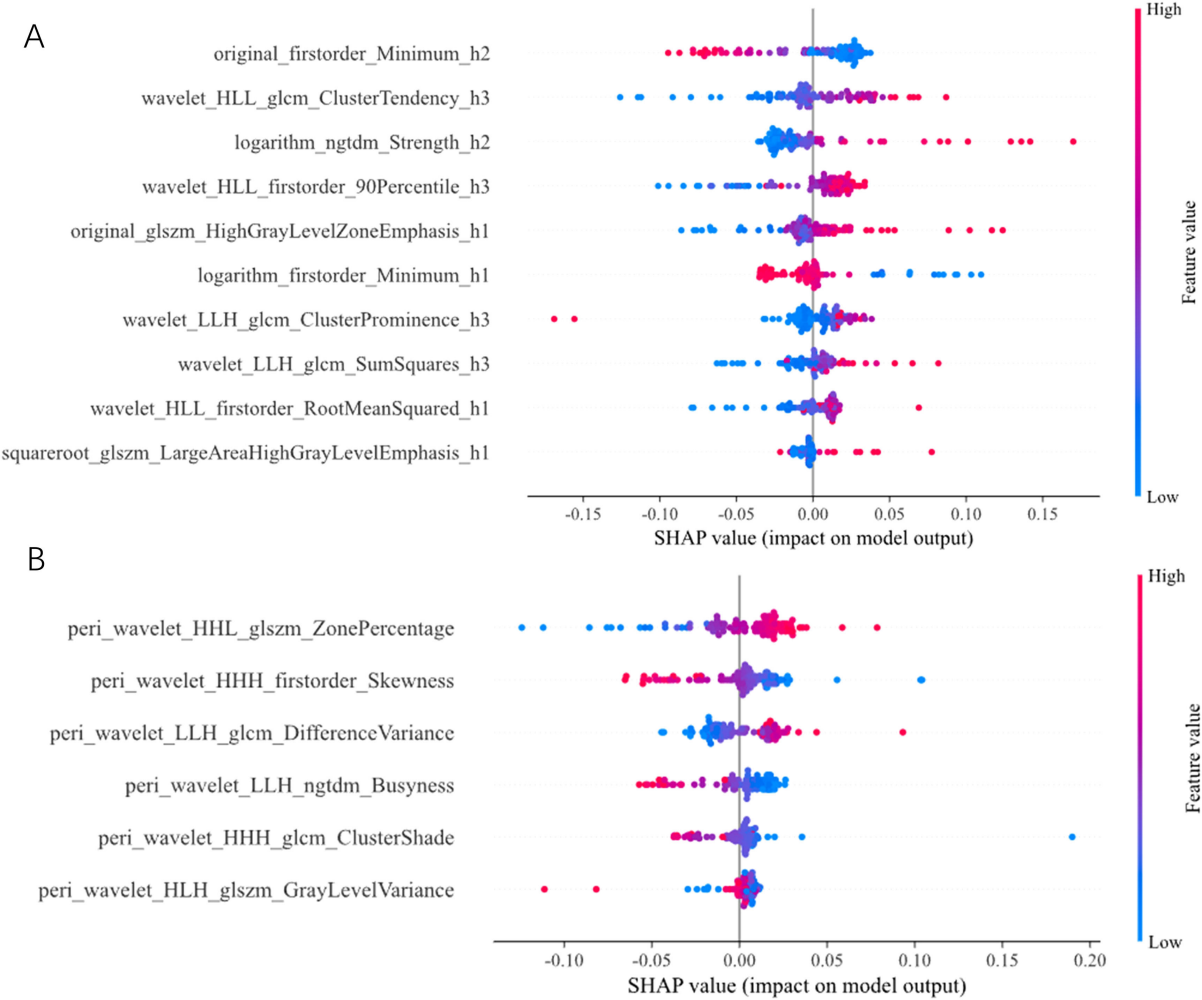
SHAP beeswarm plots of the most important radiomic features in the Habitat model **(A)** and Peri3mm model **(B)**. Features are ranked along the vertical axis in descending order of global importance. The horizontal axis represents the SHAP value (impact on model output). Dot color indicates feature value (blue: low; red: high).

**Figure 5:**
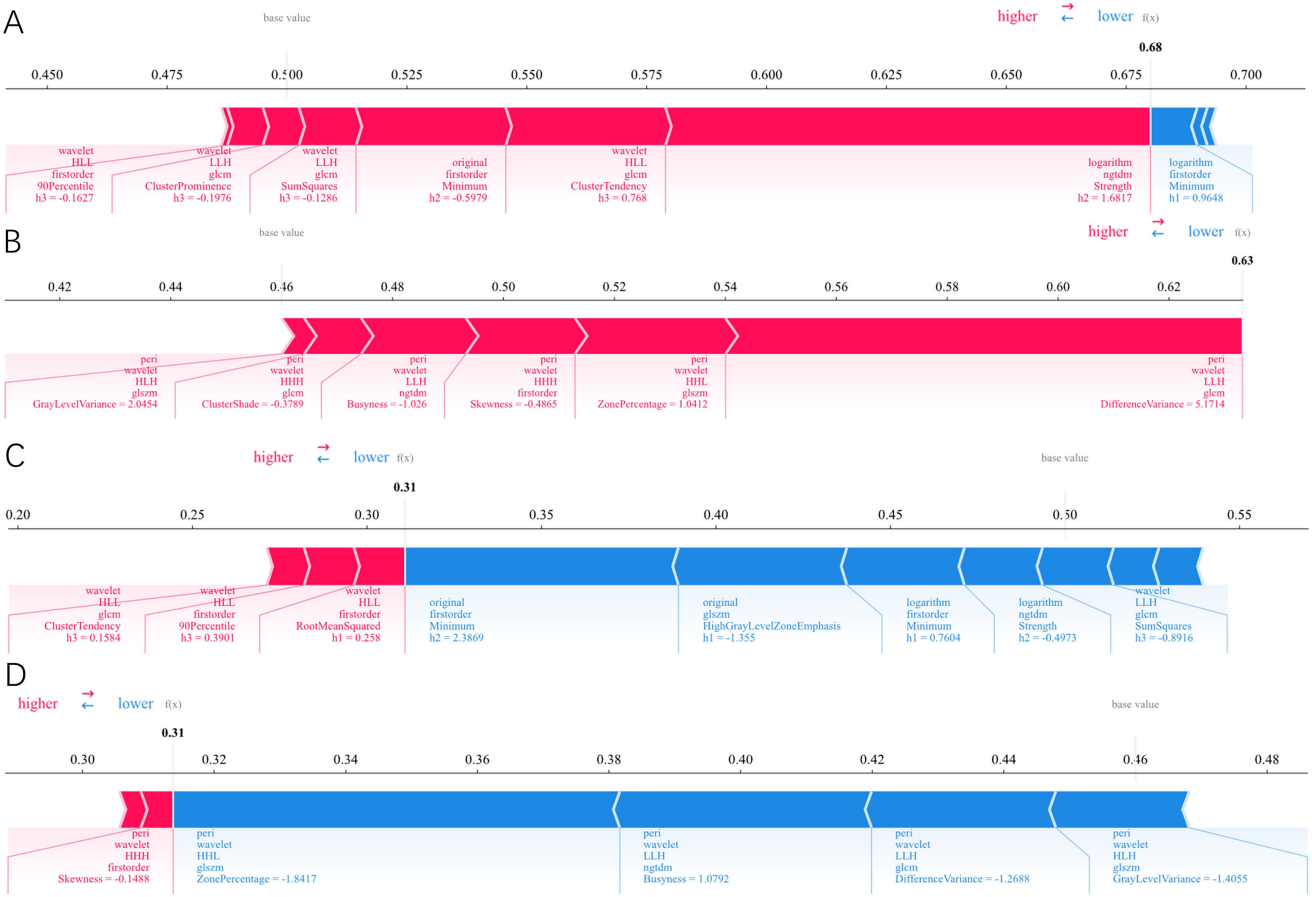
SHAP force plots are shown for one representative patient from the ORR- group and one from the ORR+ group for both the Habitat and Peri3mm models. **(A)** Habitat model prediction for Patient 1 (ORR-). The base value is 0.50, and the model’s predicted probability of non-response is 0.68. The feature logarithm_ngtdm_Strength_h2 (red) exhibits the strongest positive contribution and is the primary driver of the elevated prediction. **(B)** Peri3mm model prediction for the same Patient 1 (ORR-). The base value is 0.46, and the predicted probability of non-response is 0.63. The feature peri_wavelet_LLH_glcm_DifferenceVariance (red) demonstrates the most substantial positive contribution, dominating the positive shift in the prediction. **(C)** Habitat model prediction for Patient 2 (ORR+). The base value is 0.50, and the predicted probability of non-response is 0.31. The feature original_firstorder_Minimum_h2 (blue) provides the strongest negative contribution and serves as the key feature driving the prediction toward lower non-response risk. **(D)** Peri3mm model prediction for the same Patient 2 (ORR+). The base value is 0.46, and the predicted probability of non-response is 0.31. The feature peri_wavelet_HHL_glszm_ZonePercentage (blue) demonstrates the most prominent negative contribution, dominating the reduction in the predicted non-response probabilit**y**.

In the Habitat model (**Figure 4A**), the top five features with the highest global importance were: original_firstorder_Minimum_h2, wavelet_HLL_glcm_ClusterTendency_h3, logarithm_ngtdm_Strength_h2, wavelet_HLL_firstorder_90Percentile_h3, and original_glszm_HighGrayLevelZoneEmphasis_h1. In the Peri3mm model (**Figure 4B**), the top five globally important features were: peri_wavelet_HHL_glszm_ZonePercentage, peri_wavelet_HHH_firstorder_Skewness, peri_wavelet_LLH_glcm_DifferenceVariance, peri_wavelet_LLH_ngtdm_Busyness, and peri_wavelet_HHH_glcm_ClusterShade. To illustrate the prediction mechanism of the model at the individual level, we randomly selected one representative case from the ORR+ group and one from the ORR− group, and generated their SHAP force plots (**Figure 5**). In these plots, the SHAP value of each feature is represented by colored bars, with red indicating a positive contribution toward the model’s prediction and blue indicating a negative contribution. The length of each bar reflects the magnitude of that feature’s contribution to the overall prediction.

### 3.5 Establishment and performance of the clinical model

Univariable logistic regression analysis identified AFP as significantly associated with ORR to HTP therapy among the included clinical features (p < 0.05). Multivariable analysis (**Supplementary Table 1**) further confirmed AFP as an independent predictor of treatment response (odds ratio [OR] = 1.001, p = 0.026). Using AFP, a clinical prediction model was subsequently developed via logistic regression (LR). The model achieved AUCs of 0.725 (95% CI: 0.621–0.828) in the training cohort, 0.657 (95% CI: 0.491–0.823) in the internal validation cohort, and 0.612 (95% CI: 0.487–0.737) in the external testing cohort.

### 3.6 Evaluation of the combined model

**Figure 6:**
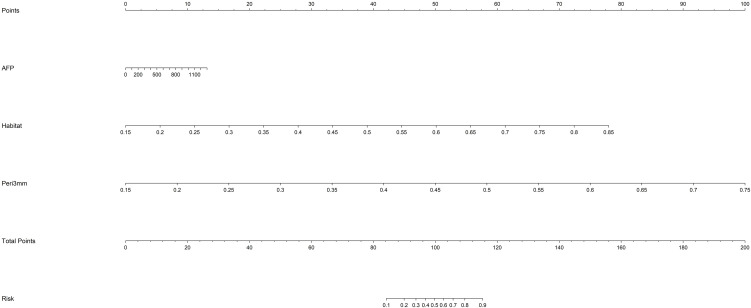
Nomogram of the combined model.

**Figure 7:**
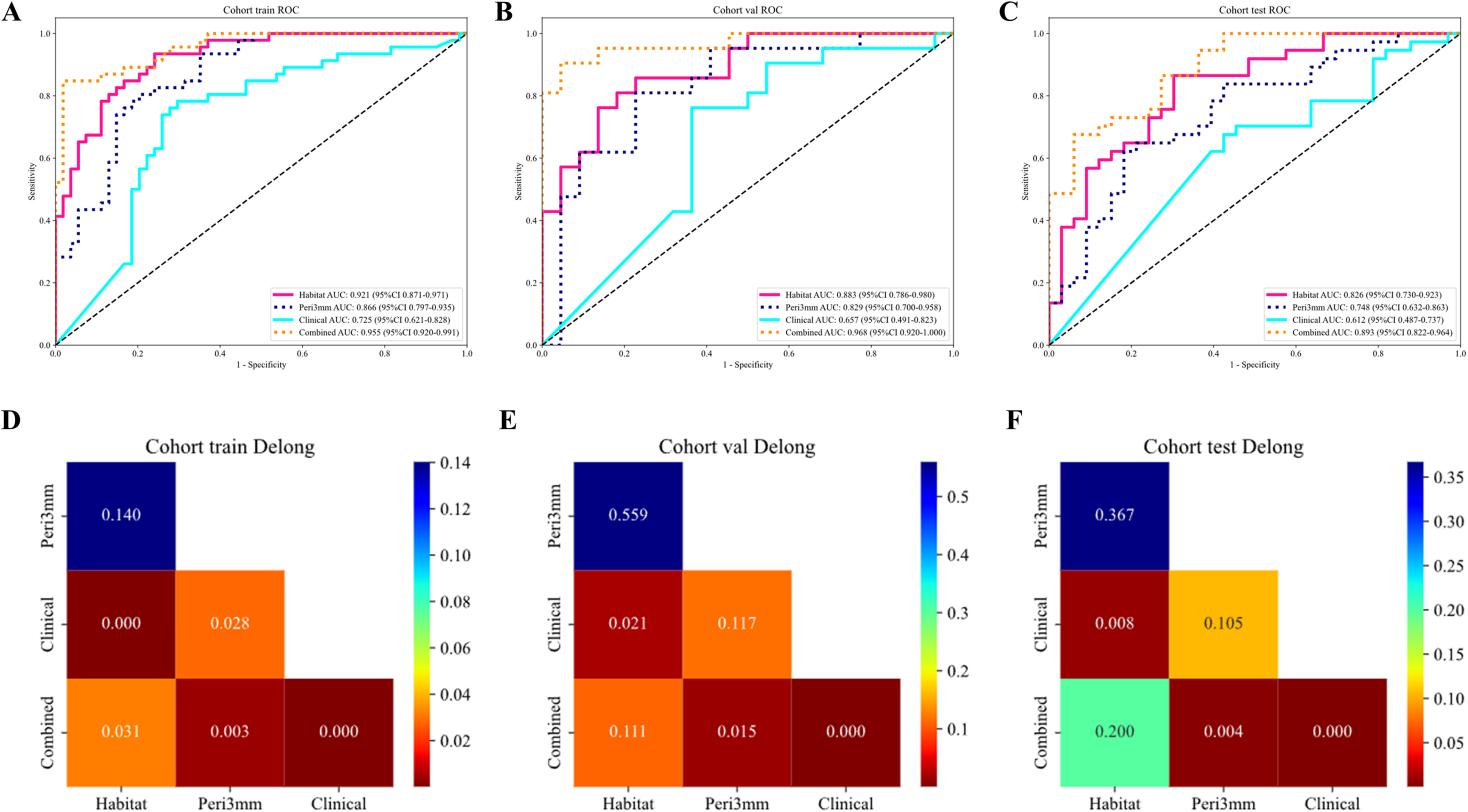
Receiver operating characteristic (ROC) curves and DeLong test results for the four key models. **(A–C)** ROC curves in the training, internal validation, and external testing cohorts, respectively. **(D–F)** Corresponding DeLong test results.

**Figure 8:**
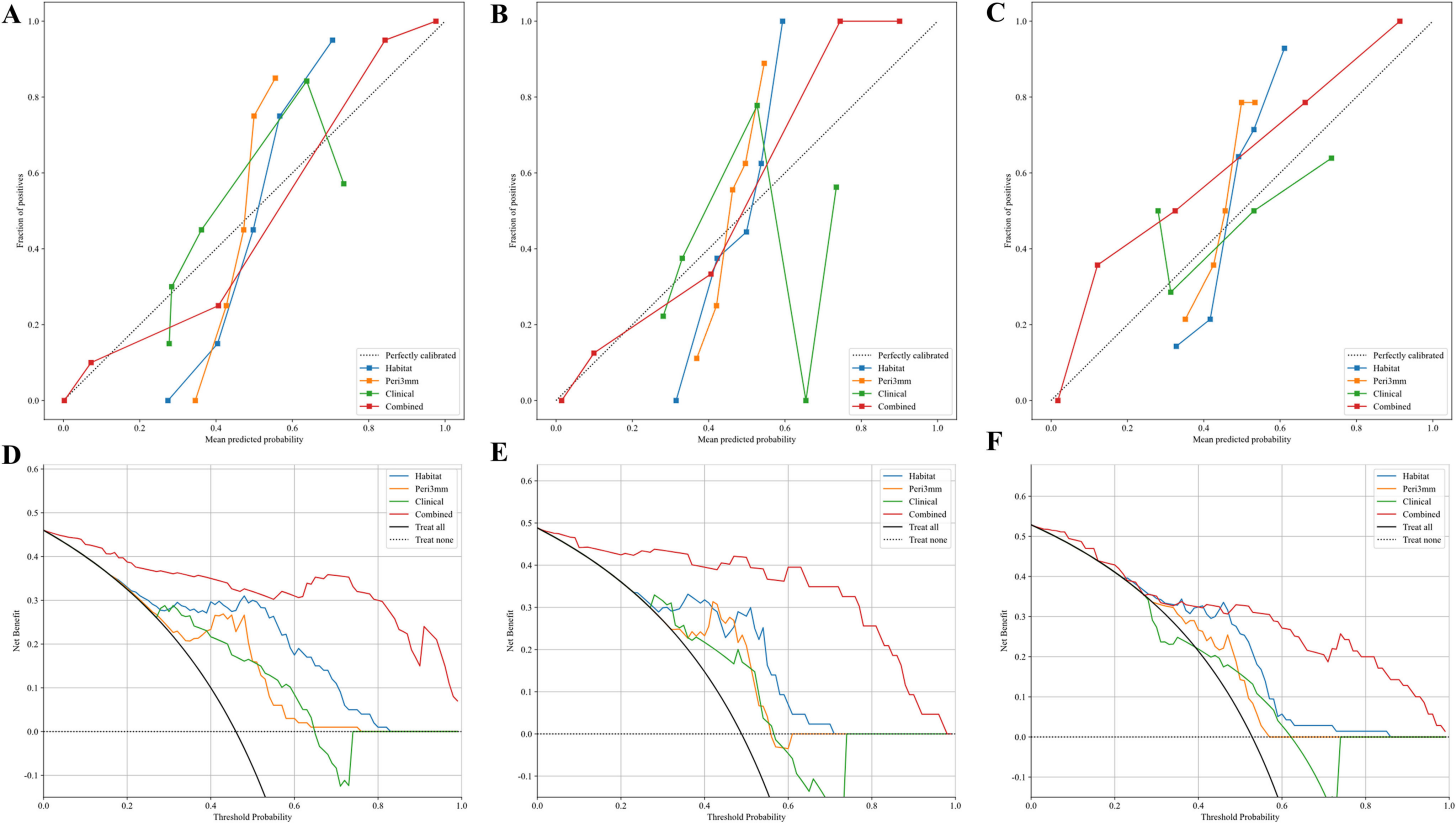
Calibration curves and decision curve analysis (DCA) for the four models. **(A–C)** Calibration curves in the training, internal validation, and external testing cohorts, respectively; the diagonal dashed line represents perfect calibration. **(D–F)** DCA in the same three cohorts; the y-axis denotes net benefit, and the x-axis represents threshold probability. The Combined model is shown in red in all panels.

Based on the comparative performance of the individual models, Habitat and Peri3mm were selected for integration. The predicted probabilities from these two models, together with the predicted probability from the clinical model, were used as input features to train a logistic regression (LR) model, which constituted the final combined prediction model. To facilitate its clinical applicability, the combined model was visualized as a nomogram (**Figure 6**). The combined model exhibited superior predictive performance across the training, internal validation, and external testing cohorts, achieving AUCs of 0.955 (95% CI: 0.920–0.991), 0.968 (95% CI: 0.920–1.000), and 0.893 (95% CI: 0.822–0.964), respectively (**Figures 7A–C**). DeLong test results confirmed that, in both the internal validation and external testing cohorts, the AUC of the combined model was significantly higher than those of all individual models except the Habitat model (P < 0.05) (**Figures 7D–F**). Although the difference in AUC between the combined model and the Habitat model did not reach statistical significance, the combined model showed improvements in accuracy, sensitivity, and other metrics. Notably, the integrated discrimination improvement (IDI) and net reclassification index (NRI) values for the combined model relative to each individual model, including the Habitat model, were all greater than zero, indicating enhanced discriminative ability and reclassification performance (**Supplementary Figure 4**). Regarding clinical applicability, the calibration curve of the combined model demonstrated excellent agreement between observed and predicted probabilities in both the internal validation and external testing cohorts, reflecting robust model calibration and reliable decision-making performance (**Figures 8A–C**). Decision curve analysis (DCA) further confirmed that the combined model provided a greater net clinical benefit across a range of threshold probabilities (**Figures 8D–F**).

### 3.7 Kaplan–Meier analysis for PFS

**Figure 9:**
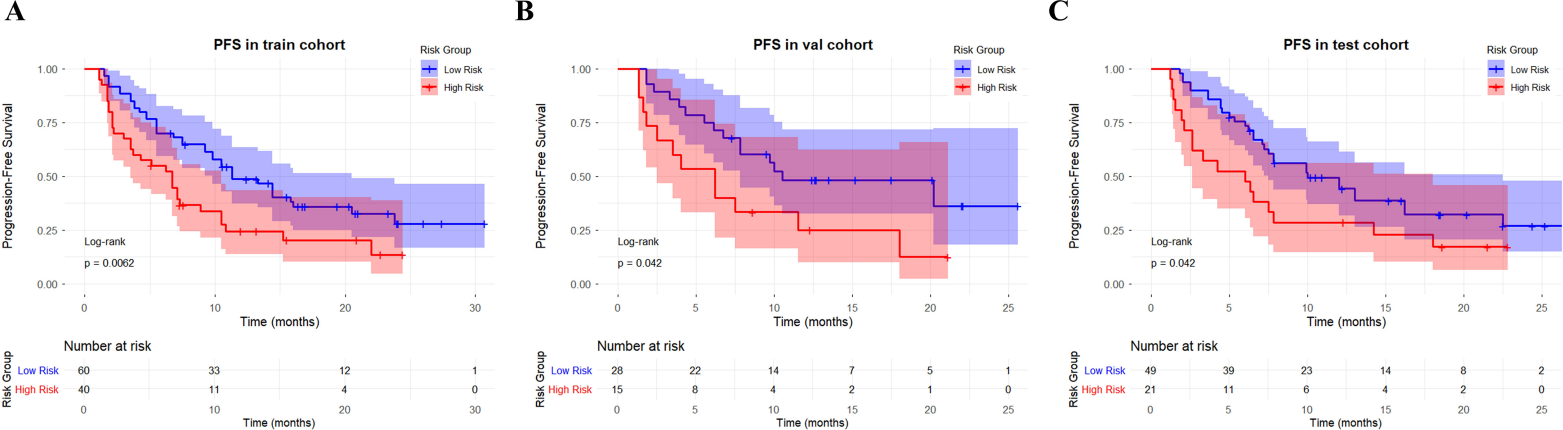
Kaplan–Meier curves of progression-free survival (PFS) stratified by the combined model risk score in the training cohort **(A)**, internal validation cohort **(B)**, and external testing cohort **(C)**.

Using the optimal cutoff value (0.677) derived from the maximum Youden index method in the training cohort, the combined model stratified patients into high-risk and low-risk groups. Across all three cohorts, high-risk patients had significantly shorter progression-free survival (PFS) than low-risk patients: training cohort (6.7 vs. 11.3 months), internal validation cohort (6.2 vs. 10.5 months), and external testing cohort (6.0 vs. 10.0 months). The differences were statistically significant (log-rank P = 0.0062, 0.0417, and 0.0419, respectively; **Figures 9A–C**).

## 4 Discussion

The pronounced heterogeneity of HCC critically determines patient responses to HTP therapy. Consequently, the precise identification and quantification of this heterogeneity are essential for advancing personalized treatment strategies. In this study, we developed a multidimensional integrated predictive model that demonstrated favorable and stable performance across the training, internal validation, and external testing cohorts, thereby offering a potential non-invasive tool for the individualized prediction of treatment response to HTP therapy.

In recent years, radiomics has been widely applied in HCC research. However, most studies still treat the entire tumor as a homogeneous entity, failing to fully capture the spatial heterogeneity arising from intratumoral blood supply and the immune microenvironment, which may limit both predictive performance and biological interpretability. For instance, Zhao et al. constructed a nomogram based on whole-tumor MRI radiomics and the ALBI score, achieving AUCs of 0.79 and 0.75 in the training and validation cohorts, respectively. Hua et al., also using whole-tumor features, developed a clinical radiomics model for lenvatinib plus PD-1 inhibitors and interventional therapy, attaining improved AUCs of 0.900 and 0.893, yet still relying on conventional intratumoral features (23, 24). Against this backdrop, habitat imaging has emerged as a promising strategy to address these limitations. Using the Calinski–Harabasz index, we partitioned the tumor into three phenotypically distinct subregions. The resulting habitat model consistently outperformed the conventional intratumoral (INTRA) model across all cohorts, underscoring the value of habitat imaging in quantifying intratumoral spatial heterogeneity.

In this study, Habitat 2 exhibited a semicircular configuration enveloping the main tumor mass, and its imaging phenotype closely corresponded to the “enhancing capsule” sign defined by the LI-RADS criteria (25). SHAP analysis suggested that two features derived from this region—original_firstorder_Minimum_h2, reflecting overall gray-level intensity, and logarithm_ngtdm_Strength_h2, representing textural homogeneity—carried substantial predictive weight. It was observed that patients with a higher risk of non-response more frequently had lower original_firstorder_Minimum_h2 values and higher logarithm_ngtdm_Strength_h2 values. From an imaging perspective, a lower Minimum_h2 value might be associated with focal segments of even lower gray-level intensity within Habitat 2, which could potentially manifest as localized decreased enhancement or interrupted signal intensity along the rim. A higher Strength_h2 value might be linked to greater deviation between local pixel intensities and the neighborhood average gray level within the subregion, which could possibly result in heterogeneous texture distribution and pronounced local contrast differences. Thus, it might be speculated that the pattern of these two features tentatively suggests a peritumoral rim with disrupted continuity and heterogeneous texture.

The peritumoral region is increasingly recognized as a dynamic and information-rich interface, whose imaging phenotype may not only harbor key features related to tumor growth, invasion, and metastasis but also reflect multiple pathophysiological processes, including microvascular invasion, inflammatory activation, and lymphangiogenesis (26, 27). While prior habitat-based investigations in HCC have centered primarily on intratumoral heterogeneity (28, 29) —exemplified by the CT-based radiomics model of Wu et al., which demonstrated predictive utility yet omitted peritumoral information entirely (30) —the present study systematically evaluated 3 mm and 9 mm peritumoral expansions. Notably, the 3 mm model achieved an AUC of 0.748 in the external testing cohort, outperforming the 9 mm model (AUC = 0.651); moreover, incorporating the Peri3mm model improved the combined model’s predictive accuracy for HTP treatment response. This discrepancy may reflect the 3 mm region’s greater sensitivity to early pathological alterations at the tumor–host interface—such as micro-infiltration and desmoplastic reaction—whereas the 9 mm expansion dilutes tumor-specific signals with normal hepatic parenchyma. This observation aligns with reports by Zhao, Kim et al., who demonstrated that 3 mm peritumoral imaging features confer significant predictive value in combined models (31, 32). Collectively, these findings reinforce the notion that the immediate peritumoral margin constitutes a critical imaging window for evaluating local invasiveness and therapeutic susceptibility in HCC.

AFP is a well-established serological biomarker in HCC and is frequently incorporated as an independent predictor in clinical models, given its general association with enhanced tumor proliferation, invasiveness, and unfavorable prognosis (33, 34). In the present study, however, the clinical model based solely on AFP exhibited limited predictive performance, yielding AUCs of 0.657 and 0.612 in the internal validation and external testing cohorts, respectively. This modest performance may be attributed to the inherent limitations of AFP as a standalone biomarker: approximately 30% of patients with HCC present with normal or low serum AFP levels, and its concentration is susceptible to confounding by non-neoplastic factors such as liver function status and hepatitis activity. Consequently, AFP alone is insufficient to capture the full spectrum of intratumoral biological heterogeneity or the characteristics of the local tumor microenvironment (35). Nevertheless, when integrated with habitat-derived and peritumoral imaging features in the combined model, AFP demonstrated significant complementary value. These findings underscore the importance of combining clinical indicators with spatially informative radiomic features to establish a more comprehensive and robust assessment framework.

In summary, although various radiomics-based models have been developed to predict treatment response in unresectable HCC, most have relied on a single technique—such as conventional intratumoral radiomics, habitat imaging, or deep learning (23, 24, 30, 36). In contrast, the present study adopted a multidimensional approach integrating habitat radiomics, peritumoral radiomics, and clinical indicators. Our combined model achieved robust predictive performance in an independent external testing cohort (AUC = 0.893), and its risk stratification distinguished progression-free survival across all three cohorts (log-rank P < 0.05 for each). Furthermore, SHAP-based interpretability analysis provided transparent insights into the contribution of individual features, partially addressing the “black-box” limitation common to many machine learning models. These findings suggest that, beyond predicting early radiologic response, the model may offer complementary information for identifying patients at higher risk of disease progression, and could support individualized surveillance and subsequent therapeutic decision-making.

Nevertheless, several limitations of this study should be acknowledged. First, although patients from two centers were included, the sample size remains relatively modest, and the retrospective design is inherently susceptible to selection bias. Prospective validation through large-scale, multicenter cohorts is therefore warranted. Second, despite our efforts to standardize imaging parameters and voxel size, advanced harmonization methods such as ComBat were not applied to correct for inter-platform CT variability. Future studies should consider adopting such methods to mitigate cross-center heterogeneity in image acquisition. Third, feature extraction was restricted to portal venous phase contrast-enhanced CT images; multiphase information (e.g., arterial or delayed phases) was not incorporated. Future research should explore the potential of multiphase image fusion to enhance predictive performance. Fourth, due to the lack of histopathological specimens, the association between the identified habitat subregions and pathological phenotypes remains to be elucidated. Therefore, these interpretations should be regarded as imaging-based hypotheses, and future imaging-pathological correlation studies are needed for validation. Fifth, owing to the consensus-based segmentation strategy, formal inter-reader agreement analysis was not performed. Finally, residual confounding from dose modifications and subsequent treatment adjustments cannot be fully excluded. Future prospective studies with more homogeneous therapeutic cohorts are warranted to further validate these findings.

## Data Availability

The datasets presented in this article are not readily available due to institutional ethics restrictions and patient confidentiality requirements. Requests to access the datasets should be directed to the corresponding author, subject to ethical approval. Requests to access these datasets should be directed to lipeizhi@hospital.cqmu.edu.cn.
