# Supplementary figures and images for: Habitat and peritumoral CT radiomics accurately predict early treatment response to hepatic arterial infusion chemotherapy combined with tyrosine kinase inhibitors and programmed death−1 inhibitors in unresectable hepatocellular carcinoma
Source: Front Oncol. 2026 May 8;16:1820483. doi: 10.3389/fonc.2026.1820483 (PMC13193938; doi:10.3389/fonc.2026.1820483)

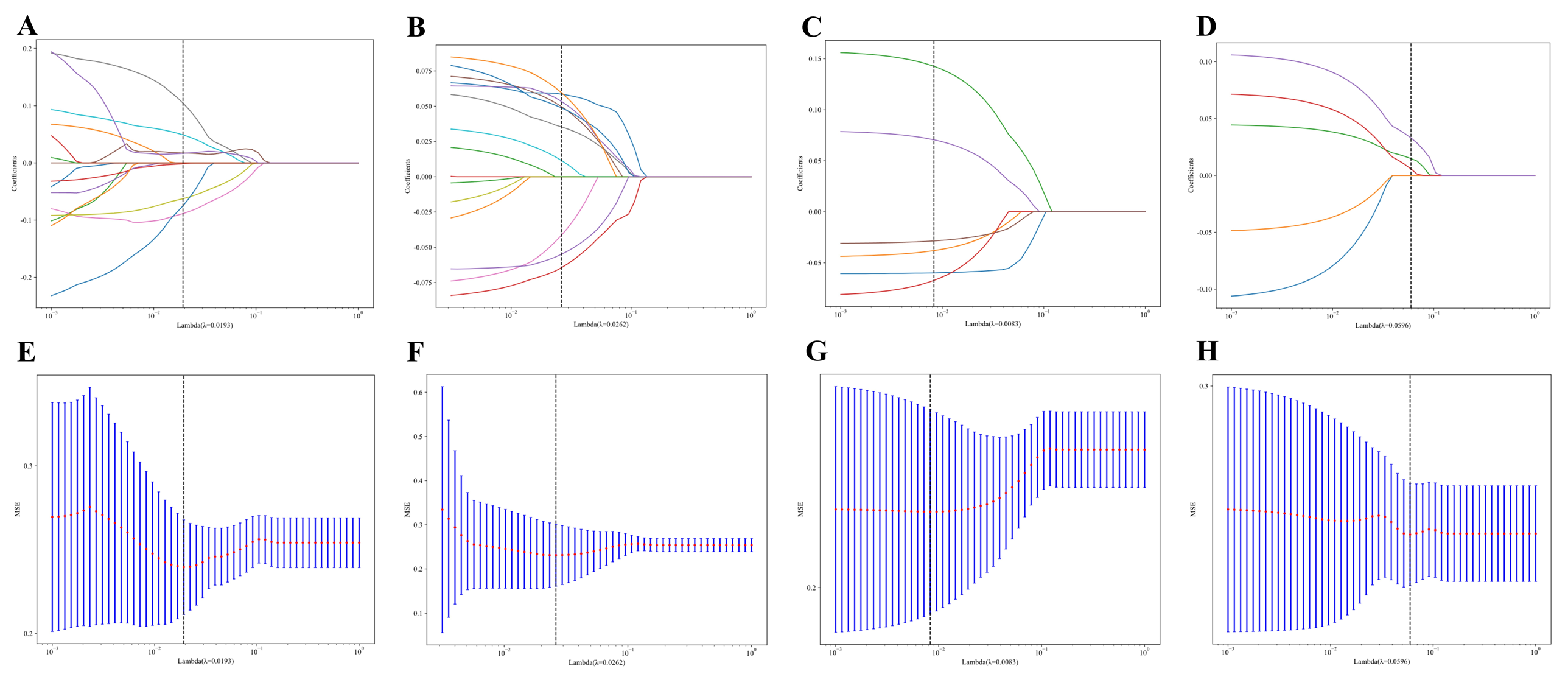

Supplement: Supplementary file 1 [file Image1.jpeg]

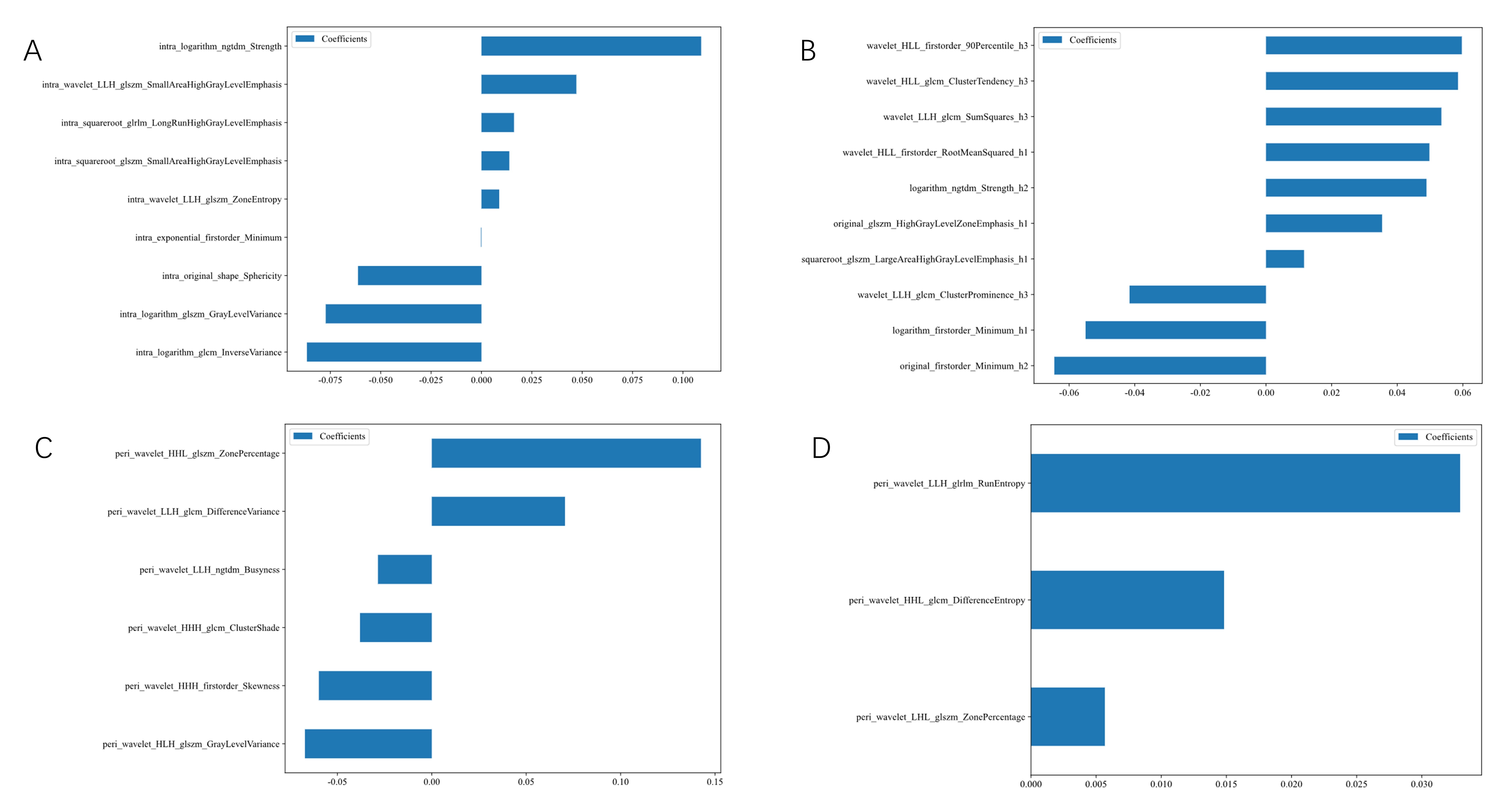

Supplement: Supplementary file 2 [file Image2.jpeg]

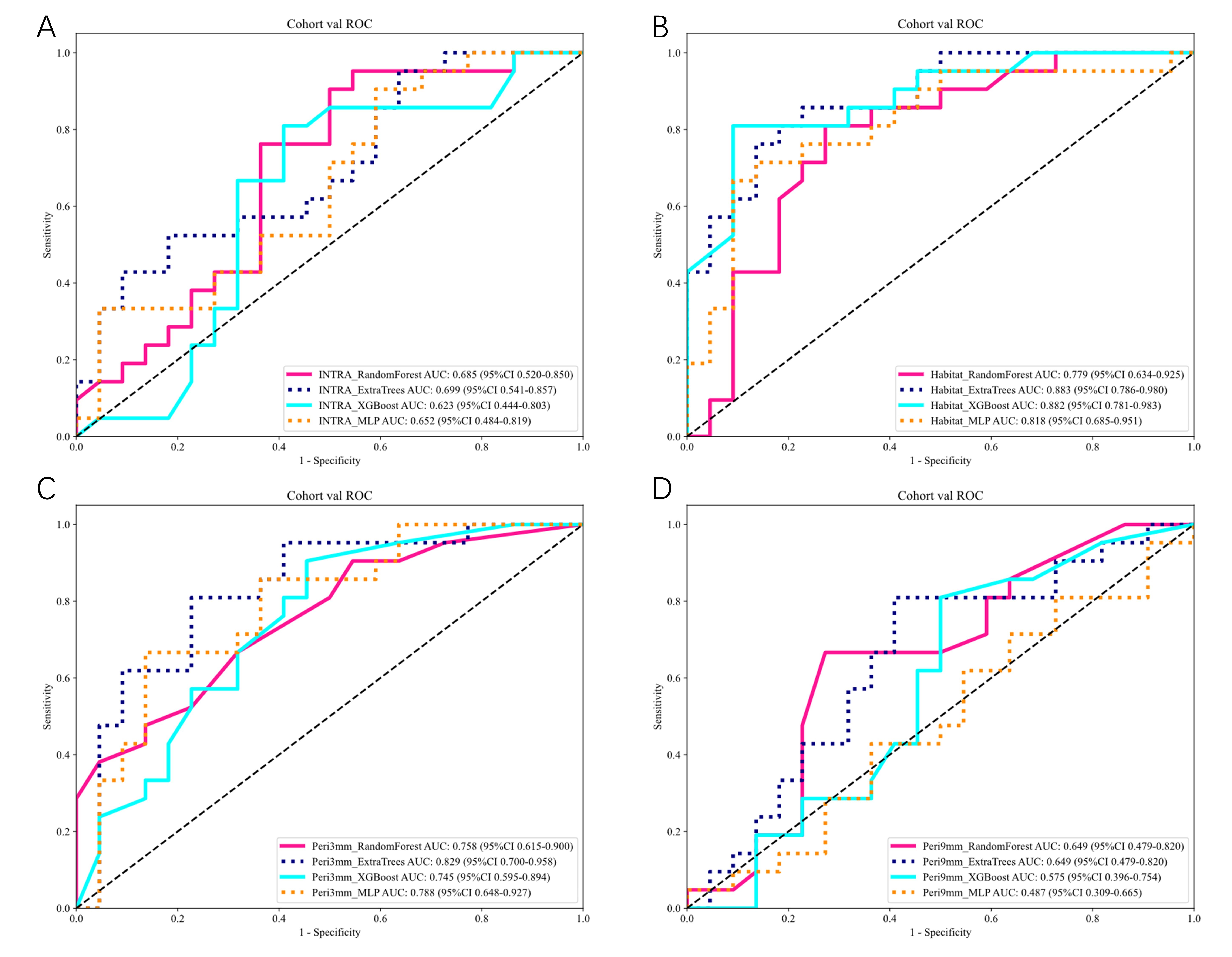

Supplement: Supplementary file 3 [file Image3.jpeg]

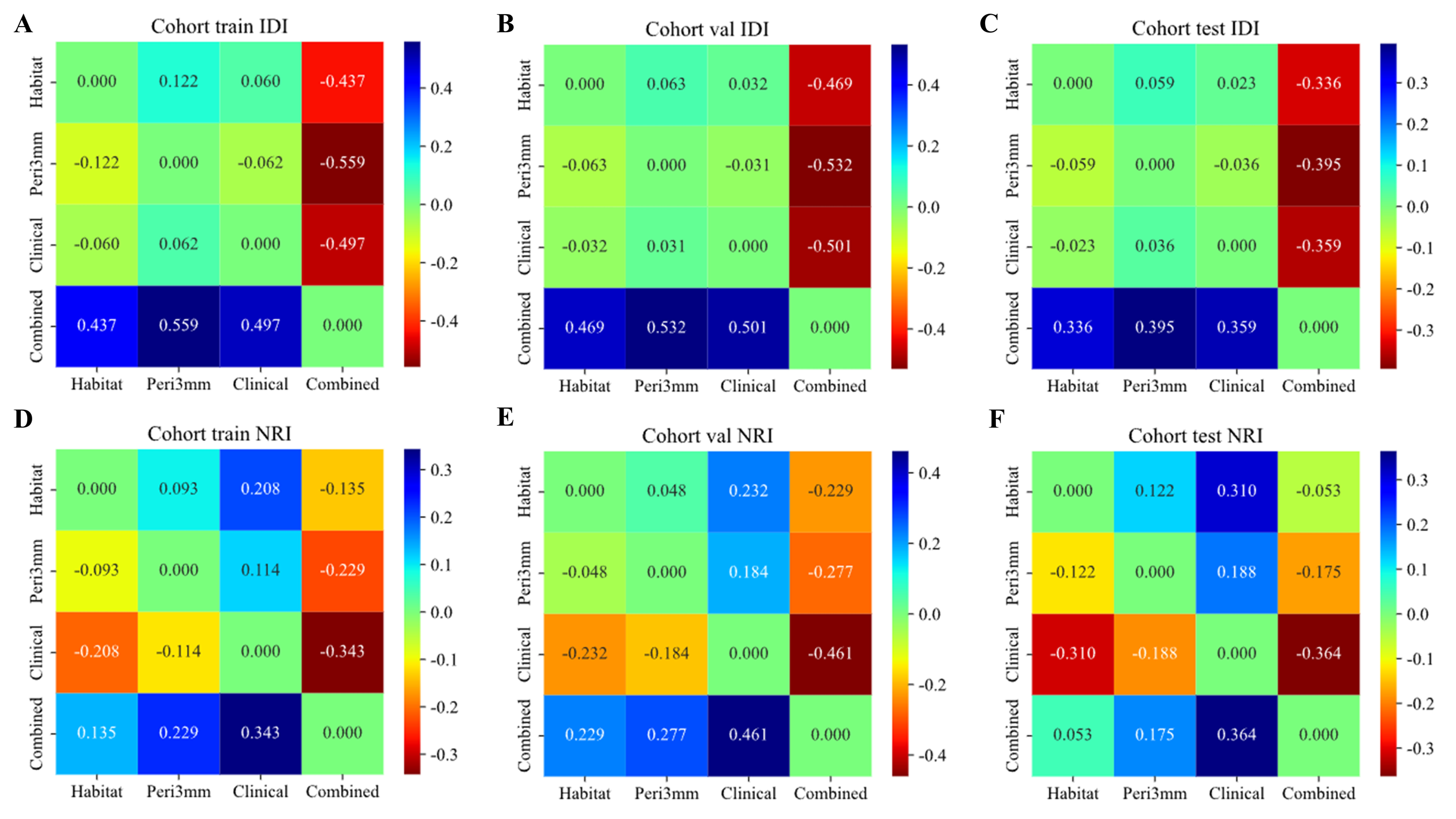

Supplement: Supplementary file 4 [file Image4.jpeg]
