# Supplementary material for: Habitat and peritumoral CT radiomics accurately predict early treatment response to hepatic arterial infusion chemotherapy combined with tyrosine kinase inhibitors and programmed death−1 inhibitors in unresectable hepatocellular carcinoma
Source: Front Oncol. 2026 May 8;16:1820483. doi: 10.3389/fonc.2026.1820483 (PMC13193938; doi:10.3389/fonc.2026.1820483)
Supplement: Supplementary file 5 [file Table1.docx]

Supplementary Material

# Supplementary Data

19 Local Features

1. First-Order Features

Entropy: Describes the uncertainty of gray level values in the image.

Mean Absolute Deviation: Represents the mean absolute deviation of gray level values in the image.

Median: Represents the median of gray level values in the image.

2. Gray-Level Co-occurrence Matrix (GLCM)

Difference Average: Represents the average of gray level differences in the image.

Difference Entropy: Represents the entropy of gray level differences in the image.

Difference Variance: Represents the variance of gray level differences in the image.

Joint Energy: Represents the energy feature of the GLCM.

Joint Entropy: Represents the entropy feature of the GLCM.

Inverse Variance: Represents the inverse variance feature of the GLCM.

Sum Entropy: Represents the entropy of gray level sums in the image.

Imc1: Represents the first information measure of correlation of the GLCM.

Imc2: Represents the second information measure of correlation of the GLCM.

3. Gray-Level Run Length Matrix (GLRLM)

Run Entropy: Represents the entropy of gray level runs.

Run Variance: Represents the variance of gray level runs.

Long Run Emphasis: Represents the emphasis on long runs.

4. Gray-Level Size Zone Matrix (GLSZM)

Size Zone Non-Uniformity Normalized: Represents the normalized non-uniformity of large zones.

Small Area High Gray Level Emphasis: Represents the emphasis on high gray-level values in small areas.

5. Neighboring Gray-Tone Difference Matrix (NGTDM)

Contrast: Represents the contrast of the image.

Strength: Represents the strength of the image.

# Supplementary Figures and Tables

## Supplementary Figures

## Supplementary Figure S1. LASSO feature selection for the four radiomics models. Coefficient profiles (upper panels) and cross-validation MSE curves (lower panels) are shown for INTRA (A, E), Habitat (B, F), Peri3mm (C, G), and Peri9mm (D, H). Vertical dashed lines indicate the optimal λ value.

**Supplementary Figure S2.** The bar plots display the features ultimately selected by LASSO regression analysis and their corresponding coefficients for the four radiomics models: (A) INTRA, (B) Habitat, (C) Peri3mm, and (D) Peri9mm.

**Supplementary Figure S3.** Receiver operating characteristic (ROC) curves of different machine learning algorithms for the four radiomic models in the internal validation cohort. (A) INTRA model; (B) Habitat model; (C) Peri3mm model; (D) Peri9mm model.

**Supplementary Figure S4.** Heatmaps of the IDI and NRI for the different models in the training, internal validation, and external testing cohorts. (A–C) IDI heatmaps; (D–F) NRI heatmaps. IDI, integrated discrimination improvement; NRI, net reclassification index.

## Supplementary Tables

| **Characteristics** | **Univariable analysis** | | **Multivariable analysis** | |
| --- | --- | --- | --- | --- |
|  | **OR (95%CI)** | ***P* value** | **OR (95%CI)** | ***P* value** |
| Ascites | 0.5 (0.245-1.019) | 0.109 |  |  |
| HBV | 0.733 (0.503-1.069) | 0.176 |  |  |
| Vascular invasion | 0.76 (0.461-1.254) | 0.367 |  |  |
| Cirrhosis | 0.793 (0.501-1.255) | 0.406 |  |  |
| sex | 0.816 (0.575-1.16) | 0.341 |  |  |
| Child Pugh | 0.855 (0.654-1.116) | 0.335 |  |  |
| BCLC | 0.864 (0.71-1.05) | 0.218 |  |  |
| VP | 0.879 (0.763-1.012) | 0.133 |  |  |
| ALBI grade | 0.897 (0.74-1.088) | 0.353 |  |  |
| Ly | 0.955 (0.814-1.121) | 0.636 |  |  |
| PT | 0.986 (0.963-1.01) | 0.343 |  |  |
| Tumor size | 0.989 (0.954-1.024) | 0.603 |  |  |
| BMI | 0.995 (0.981-1.009) | 0.559 |  |  |
| Neu | 0.996 (0.94-1.055) | 0.916 |  |  |
| ALB | 0.996 (0.988-1.005) | 0.484 |  |  |
| age | 0.997 (0.992-1.003) | 0.454 |  |  |
| AST | 0.999 (0.996-1.002) | 0.484 |  |  |
| ALP | 0.999 (0.998-1.001) | 0.579 |  |  |
| PLT | 0.999 (0.997-1.001) | 0.520 |  |  |
| GGT | 1.0 (0.999-1.002) | 0.547 |  |  |
| ALT | 1.0 (0.996-1.005) | 0.931 |  |  |
| AFP | 1.001 (1.0-1.001) | 0.026 | 1.001 (1.0-1.001) | 0.026 |
| TBIL | 1.002 (0.997-1.006) | 0.508 |  |  |
| ALBI score | 1.06 (0.93-1.208) | 0.468 |  |  |
| Number of tumors | 1.074 (0.692-1.667) | 0.789 |  |  |
| Metastasis | 1.273 (0.656-2.469) | 0.549 |  |  |
| ECOG | 1.393 (0.927-2.094) | 0.181 |  |  |

**Supplementary Table S1.** Univariable and multivariable analysis of clinical features.

OR, odds ratio; CI, confidence interval; AFP, alpha-fetoprotein; ALB, albumin; ALBI, albumin-bilirubin grade; ALP, alkaline phosphatase; ALT, alanine aminotransferase; AST, aspartate aminotransferase; BCLC, Barcelona Clinic Liver Cancer stage; BMI, body mass index; GGT, gamma-glutamyl transferase; HBV, hepatitis B virus; Neu, neutrophil count; Ly, lymphocyte count; PLT, platelet count; PS, performance status; PT, prothrombin time; TBIL, total bilirubin; VP, vascular invasion.
